# Successful management of fecalith impaction in the distal ileum using a transendoscopic enteral tube for targeted drug delivery

**DOI:** 10.1055/a-2376-7350

**Published:** 2024-08-13

**Authors:** Quan Wen, Bota Cui, You Yu, Faming Zhang

**Affiliations:** 1 637622 Department of Microbiota Medicine & Medical Center for Digestive Diseases, The Second Affiliated Hospital of Nanjing Medical University, Nanjing, China; 2 637622 Key Lab of Holistic Integrative Enterology, The Second Affiliated Hospital of Nanjing Medical University, Nanjing, China

A 69-year-old man presented after 1 week of abdominal pain and distension, accompanied by nausea and vomiting for 1 day. Computed tomography identified wall thickening of the distal small intestine, with a 3.4 × 2.8 cm high-density intraluminal foreign body ( Fig. 1 ) and proximal intestinal dilation with air/fluid levels. Despite conservative treatments, including intravenous hydration, feeding tubes, stool softeners, and enema, the patient’s symptoms persisted. Intestinal ultrasound confirmed a strong echo mass in the distal ileum.

**Fig. 1:**
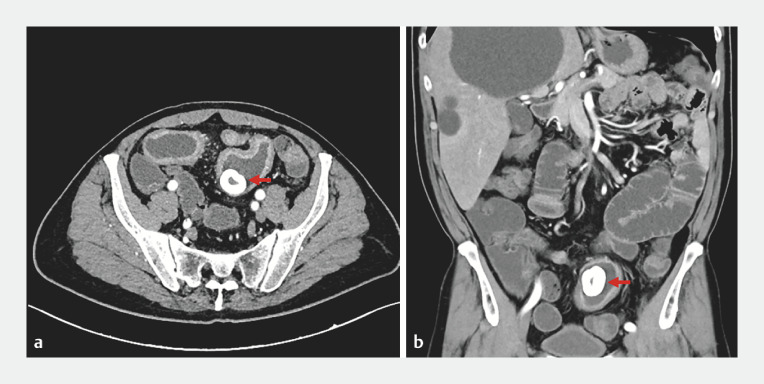
Computed tomography revealed distal small intestinal wall thickening with a 3.4 × 2.8 cm high-density intraluminal foreign body (red arrow). **a** Transverse plane. **b** Coronal plane.

Subsequently, a double-balloon enteroscopy was performed to remove the foreign body, revealing a black fecalith impacted in the distal ileum approximately 40 cm from the ileocecal valve ( Fig. 2 ). Furthermore, a circular ulcer with stenosis was observed surrounding the fecalith, complicating its removal. Despite attempts with a balloon catheter and laser lithotripsy, the fecalith could not be dislodged owing to its characteristics and intestinal stenosis. Endoscopic incision and dilation for inflammatory ulcer stenosis also involved significant risks.

**Fig. 2:**
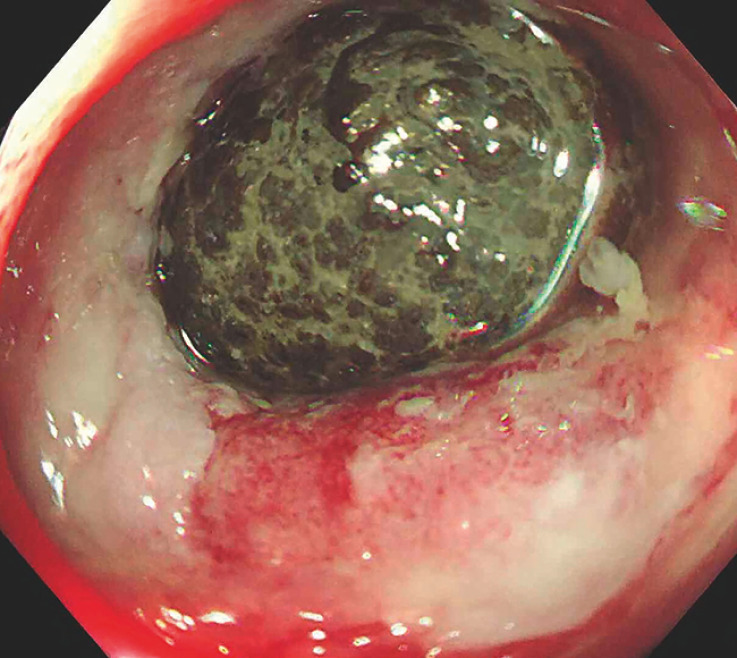
A black fecalith was impacted in the distal ileum, approximately 40 cm from the ileocecal valve, and surrounded by a circular ulcer with stenosis.

Ultimately, the transendoscopic enteral tube (TET) technique was performed below the fecalith for targeted drug delivery. Dexamethasone (10 mg/day) was administered via TET for 3 days to alleviate the inflammatory stenosis. Follow-up computed tomography revealed improvement of the distal ileum inflammation ( Fig. 3 ), and the fecalith had descended to the terminal ileum near the ileocecal valve ( Fig. 4 ). To further facilitate fecalith expulsion, Gastrografin solution (AZ Imaging, Neimenggu, China) was injected through the TET 1 . The patient successfully passed the fecalith 1 day later ( Fig. 5 ).

**Fig. 3:**
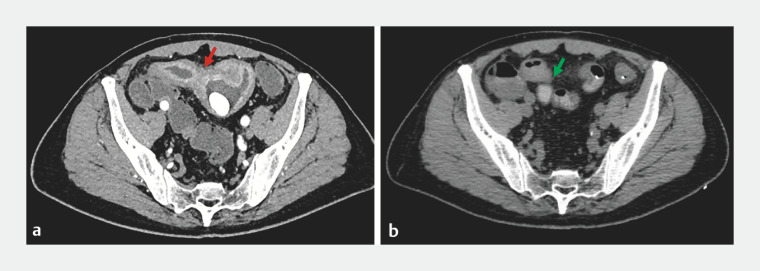
Computed tomography images of the distal ileum before ( **a** ) and after ( **b** ) treatment. The red arrow indicates the thickening and stenosis of the distal ileum wall before treatment, as well as fecalith retention above the intestinal stenosis. The green arrow shows significant improvement in inflammation of the distal ileum, with no observed thickening or stenosis of the intestinal wall, following the delivery of dexamethasone into the ileum via the transendoscopic enteral tube.

**Fig. 4:**
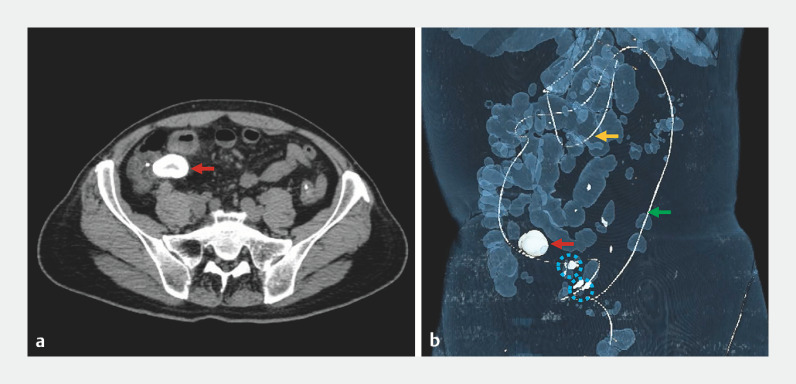
Following the targeted administration of dexamethasone into the ileum through the transendoscopic enteral tube (green arrow) to treat inflammatory stenosis, the fecalith (red arrow) was expelled through the narrowed intestine to the terminal ileum near the ileocecal valve. The blue dotted circle indicates clips fixed onto the intestinal wall. The yellow arrow shows a nasojejunal tube. **a** Computed tomography transverse plane. **b** Three-dimensional image.

**Fig. 5:**
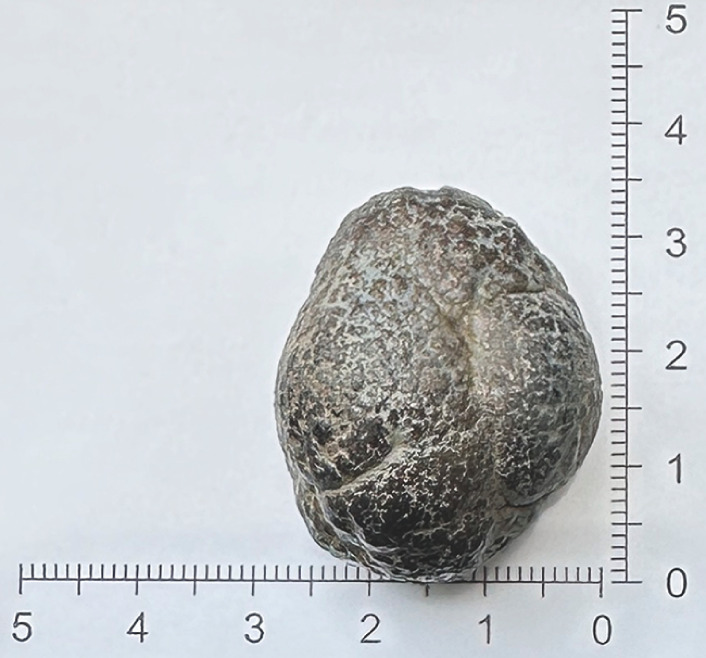
The hard fecalith from the ileum (3.4 × 2.8 cm).

Colonic TET, as an innovative technique, enables the multiple delivery of microbiota suspensions, colon-targeted drug administration, and decompression for perforation and stenosis 2 3 4 . This is the first report of targeted drug delivery to the distal ileum using TET, successfully treating distal ileal obstruction caused by fecalith impaction and thus avoiding surgical intervention ( Video 1 ). Compared with traditional drug treatments and surgical procedures, the TET intervention in the ileum may have special value.

Management of fecalith impaction in the distal ileum using a transendoscopic enteral tube for targeted drug delivery.Video 1

Endoscopy_UCTN_Code_CCL_1AC_2AH

## Notes

Endoscopy E-Videos https://eref.thieme.de/e-videos
*E-Videos* is an open access online section of the journal *Endoscopy* , reporting on interesting cases and new techniques in gastroenterological endoscopy. All papers include a high-quality video and are published with a Creative Commons CC-BY license. Endoscopy E-Videos qualify for HINARI discounts and waivers and eligibility is automatically checked during the submission process. We grant 100% waivers to articles whose corresponding authors are based in Group A countries and 50% waivers to those who are based in Group B countries as classified by Research4Life (see: https://www.research4life.org/access/eligibility/ ). This section has its own submission website at https://mc.manuscriptcentral.com/e-videos .
